# Recent Advances in Scar Biology

**DOI:** 10.3390/ijms19061749

**Published:** 2018-06-13

**Authors:** Rei Ogawa

**Affiliations:** Department of Plastic, Reconstructive and Aesthetic Surgery, Nippon Medical School, 1-1-5 Sendagi, Bunkyo-ku, Tokyo 113-0022, Japan; r.ogawa@nms.ac.jp; Tel.: +81-3-5814-6208; Fax: +81-5685-3076

Scars develop in the final stage of wound healing. The biological pathways that underlie wound healing and scarring are complex. In particular, the exact mechanisms that initiate and regulate them and lead to their progression remain to be fully elucidated. A major goal of medical science is scar-less wound healing. To achieve this goal, it is necessary to elucidate the relevant clinical, histopathological, and molecular manifestations of scars, and to understand how these manifestations relate to each other. The purpose of the Special Issue “Recent Advances in Scar Biology” that was published recently in the *International Journal of Molecular Sciences* was to illustrate the biological mechanisms that underpin scarring and effective clinical treatments. This Special Issue included a selection of recent research topics and current review articles in the field of scar research for all kinds of tissues and organs.

Normally, the cutaneous wound healing process closes skin gaps by inducing the formation of granulation tissue and epithelialization, which re-establishes an effective epidermal barrier. The complex biochemical events that underlie wound closure can be categorized into four overlapping processes: coagulation, inflammation, proliferation and remodeling. Coagulation and the inflammatory process begin immediately after injury, while the proliferative phases start within a few days. The remodeling phase commences within a week of injury and continues for months. If the inflammatory and proliferative phases are feeble, wound healing may be delayed and chronic wounds may develop. In relation to this, Horng et al. [1] showed in the Special Issue that estrogen deficiency, such as that in postmenopausal women, has detrimental effects on wound-healing processes, particularly inflammation and re-granulation, and that exogenous estrogen treatment may reverse these effects [1]. Conversely, if the inflammatory and proliferative phases are excessively vigorous and prolonged due, for example, to infection or burn, heavy scars can develop. Clinical interventions that target these phases can, therefore, improve wound healing. For example, Jeong et al. [2] showed in a rat incisional wound-healing model that injections with polydeoxyribonucleotide (a mixture of nucleotides from trout sperm) have anti-inflammatory effects and, therefore, reduce the size of the scar [2].

After full-thickness burning, necrotized tissues (eschars) develop. These eschars delay wound healing, thereby promoting the formation of hypertrophic scars. Monsuur et al. [3] showed in the Special Issue that, while acellular extracts of burn eschars stimulate the proliferation and migration of adipose mesenchymal stromal cells and fibroblasts, they also inhibit the basic fibroblast growth factor-induced proliferation and sprouting of endothelial cells. This inhibitory effect may explain why the presence of an eschar blocks the formation of excessive granulation tissue by full-thickness burn wounds [3]. Akita et al. also showed that proper epithelialization plays an important role in the healing of burn wounds: when patients with extensive burns received cultured epithelial autografts (CEA) along with either highly expanded (over 1:6 ratio) or less expanded (gap 1:6) mesh, the former combination was associated with accelerated wound healing. Moreover, scoring by experts using the Vancouver and Manchester Scar Scales showed that CEA with the highly expanded mesh led to better scar formation [4]. The exhaustive review of Mostaço-Guidolin et al. also showed that proper formation of the extracellular matrix plays a key role in the epithelialization and other wound-healing events that lead to a smooth wound-healing course: studies that used second harmonic generation microscopy to image the fibrillar collagens in wounded and repaired skin, lung, cardiovascular, tendon and ligaments, and eye tissue indicate that the balance between extracellular matrix synthesis and degradation determines the degree of scarring after wounding [5].

Multiple wound-healing processes, including granulation tissue formation and wound contraction and epithelialization, are influenced by mechanical forces [6,7]. The mechanisms by which these forces shape wound healing remain to be fully elucidated, but the review of Januszyk et al. [8] in the Special Issue suggests that focal adhesion kinase (FAK), which is a mediator of mechanotransduction pathways, plays a central role in both the inflammation and fibrosis that characterizes aberrant wound healing [8]. Moreover, multiple lines of evidence suggest that the formation of granulation tissue and numerous functions of fibroblasts, myofibroblasts, endothelial cells, and epithelial cells are affected by intrinsic and extrinsic mechanical stimuli. In recent years, many mechanosensors in these cells and tissues and the mechanosignaling pathways that they trigger have been elucidated [9,10,11]. These mechanosensors include mechanosensitive ion channels, cell-adhesion molecules (including integrins), and actin filaments in the cytoskeleton. When these structures and molecules sense mechanical stimuli, signaling pathways are activated and gene expression is altered. An important family of mechanosensitive ion channels is the transient receptor potential cation channel (TRP channel) family. Its members include TRP vanilloid (TRPV) 4, which is a mechanosensor in the skin [9], and TRPV3, which is a temperature sensor and vasoregulator. Park et al. reported that TRPV3 may contribute to the pruritus in burn scars by increasing the expression of thymic stromal lymphopoietin by epidermal keratinocytes. Thymic stromal lymphopoietin is a cytokine that has been linked to allergic and fibrotic diseases. Thus, thymic stromal lymphopoietin may be a potential therapeutic target for post-burn pruritus [12]. In relation to the signaling pathways that are triggered by mechanosensors, one may be the transforming growth factor (TGF)-β/SMAD pathway. This pathway plays a very well known role in collagen synthesis and fibrosis, but several lines of evidence suggest that it is also a mechanosignaling pathway [10,11]. This is supported by the study of Maeda et al., who subjected canine eyes to, first, glaucoma filtration surgery and, then, subconjunctival implantation of gelatin hydrogel with and without an anti-TGF-β antibody. They found that the controlled release of the anti-TGF-β antibody was associated with better intraocular pressure and less bleb formation and conjunctival scarring [13]. Other important mechanosignaling pathways are the mitogen-activated protein kinase (MAPK) and NF-κB interaction signaling pathways [10].

In relation to cutaneous scarring specifically, keloids and hypertrophic scars develop when the inflammation process is prolonged. Common initiators of these scars are cutaneous injury (including trauma) and irritation, insect bites, burn, surgery, vaccination, skin piercing, acne, folliculitis, chicken pox, and herpes zoster infection. These injuries and infections appear to result in chronic inflammation of the reticular layer of the dermis, which then drives the aberrant growth of keloids and hypertrophic scars [14]. The involvement of the reticular dermal layer is crucial: superficial injuries that do not reach the reticular dermis never cause these heavy scars. Reticular dermal inflammation may be promoted by a number of external and internal post-wounding stimuli, including mechanical tension on the wound edge, systemic factors such as sex hormones, and genetic factors [14]. Several molecules that play important roles in excessive cutaneous scarring have been identified. Kim et al. reported in this Special Issue that one of these may be high-mobility group box 1 (HMGB1): when normal and keloid fibroblasts were treated with HMGB1 or its inhibitor, their migration was accelerated and inhibited, respectively [15]. Moreover, Yamawaki et al. reported that the serine protease HtrA1 not only participates in the development of diseases such as osteoarthritis and age-related macular degeneration, it may also play an important role in keloid pathogenesis: they showed that keloid tissue fibroblasts express higher levels of this protein than surrounding normal skin and that silencing HtrA1 expression inhibits keloid fibroblast proliferation [16].

Numerous preventive and treatment strategies for keloids and hypertrophic scars have been reported. They include corticosteroid injection/tape/ointment, radiotherapy, cryotherapy, compression therapy, stabilization therapy, 5-fluorouracil (5-FU) therapy, and surgical methods [17,18,19]. In their review in this Special Issue, Lee et al. [20] summarized these methods after describing the wound-healing phases, the proteins and cytokines that play important roles in each phase, and some recently discovered anti- and pro-fibrotic pathways (e.g., hypoxia) [20]. Moreover, Park et al. reported that −79 °C spray-type cryotherapy effectively treats keloids [21]. Similarly, Cui et al. [22] reported that extracorporeal shock-wave therapy markedly improves the appearance and symptoms of post-burn hypertrophic scars, apparently by inhibiting the epithelial–mesenchymal transition [22].

Thus, the Special Issue “Recent Advances in Scar Biology” that was published in the *International Journal of Molecular Sciences* provides intriguing glimpses into the current wound healing/scarring field. It will be of interest for researchers and physicians who wish to understand the mechanisms that underlie wound healing and scarring and how these mechanisms can be manipulated to yield effective treatments of wounds and scars.
